# GATA6 Facilitates Progression of Intervertebral Disc Degeneration by Regulating Ferroptosis via Targeting TLR2/AKR1C3

**DOI:** 10.7150/ijbs.102776

**Published:** 2025-01-13

**Authors:** Xiaobo Wang, Bingyu Wang, Jiawei Shi, Zefu Chen, Yongpei Wu, Jingmin Liu, Zhongmin Zhang, Yang Wang, Jia Dan, Xin Zheng

**Affiliations:** 1 Department of Spine Surgery, Nanfang Hospital, Southern Medical University, Guangzhou, China.; 2 Department of Spine Surgery, The Affiliated TCM Hospital of Guangzhou Medical University, Guangzhou, Guangzhou, China.; 3 Organ Transplant Center, the First Affiliated Hospital, Sun Yat-Sen University, Guangzhou, China.; 4 Department of Radiology, Zhujiang Hospital, Southern Medical University, Guangzhou, China.; 5 Department of Orthopaedics, the First Affiliated Hospital, Zhejiang University School of Medicine, Hangzhou, China.

**Keywords:** intervertebral disc degeneration, nucleus pulposus, ferroptosis, GATA6, TLR2, AKR1C3

## Abstract

This study explored the role of ferroptosis in intervertebral disc degeneration (IVDD), and identified GATA6 as a key regulator of this process. A ferroptosis-related gene risk coefficient model was constructed using differential gene expression analysis of the GSE70362 dataset. The analysis identified GATA6 as a significant factor in IVDD progression. GATA6 was shown to promote ferroptosis in nucleus pulposus cells (NPCs) by regulating the expression of AKR1C3 through the TLR2 pathway. *In vitro and in vivo* experiments demonstrated that GATA6 knockdown reduced ferroptosis, improved cell viability, and mitigated extracellular matrix degradation, whereas GATA6 overexpression exacerbated these processes. Furthermore, AKR1C3 was found to be crucial for GATA6-mediated ferroptosis, and modulation of the TLR2-AKR1C3 axis significantly impacted the degeneration of NPCs. These findings suggest that targeting GATA6 and its downstream TLR2-AKR1C3 pathway may provide new therapeutic approaches for IVDD.

## Introduction

The intervertebral disc (IVD) is composed of three distinct components: the gel-like nucleus pulposus (NP), the fibrocartilaginous annulus fibrosus, and the cartilaginous endplates, all of which collectively confer flexibility and structural integrity to the spine 1. Intervertebral disc degeneration (IVDD) is a prevalent musculoskeletal disorder, primarily characterized by a reduction in intervertebral space height and a decrease in NP hydration, which results from proteoglycan loss and the functional impairment of nucleus pulposus cells (NPCs) 2. IVDD is widely recognized as a leading cause of lower back pain, neurological deficits, and pain radiating to the buttocks, legs, and neck 3, 4. These clinical manifestations place a significant socioeconomic burden on approximately 80% of the global population at some point in their lives 5. However, the molecular mechanisms underlying IVDD remain poorly understood, with its precise pathogenesis yet to be fully elucidated.

Recent research has identified the inflammatory microenvironment within the disc as a critical factor to pathogenesis of IVDD 6-9. Elevated levels of pro-inflammatory mediators within the IVD initiate a cascade of pathological events, including the generation of reactive oxygen species (ROS), degradation of the extracellular matrix (ECM), and various forms of programmed cell death, such as apoptosis, necroptosis, and ferroptosis—the latter defined by iron-dependent lipid peroxidation 10, 11. Ferroptosis has been shown to be a key process in IVDD as it accelerates degeneration by promoting immune cell infiltration. Disrupted iron metabolism and impaired glutathione peroxidase 4 (GPX4) function, which is often triggered by activation of the mechanosensitive Piezo1 channel, can further exacerbate ferroptosis in NPCs and increase the progression of IVDD 12. Efforts to stabilize the interaction between ubiquitin-specific peptidase 11 (USP11) and sirtuin 3 (Sirt3) have shown promise in mitigating ferroptosis induced by oxidative stress, thereby offering a potential therapeutic approach for IVDD 13. Additionally, the use of polydopamine nanoparticles to target ferroptosis has demonstrated encouraging results in preclinical models, suggesting their utility in treating IVDD 14. Collectively, these findings indicate that inhibition of ferroptosis could serve as a viable therapeutic strategy to slow or halt the progression of IVDD, and thus, warranting further research to elucidate the precise molecular mechanisms involved.

To investigate the key molecular drivers of ferroptosis in NPCs and their contribution to IVDD, we developed a genetic risk model focused on ferroptosis, and identified GATA-binding protein 6 (GATA6) as a pivotal factor linking ferroptosis to the progression of IVDD. GATA6 is a zinc-finger transcription factor that plays an essential role in cell differentiation, development, and metabolic regulation 15-17. Notably, GATA6 exhibits a dual function as both an oncogene and tumor suppressor 18, with the additional capacity to protect cells from DNA damage related to cell proliferation 19. However, its involvement in IVDD and its regulatory effect on ferroptosis has not been fully characterized. In this study, we demonstrated that silencing GATA6 in NPCs significantly inhibited ferroptosis and attenuated IVDD progression, as evidenced by reduced degradation of the ECM. Further mechanistic exploration revealed that GATA6 induces ferroptosis by upregulating the expression of Aldo-keto reductase family 1 member C3 (AKR1C3) via the Toll-like receptor 2 (TLR2) pathway. AKR1C3, which plays a role in steroid metabolism and prostaglandin synthesis, has been shown to regulate ferroptosis in hepatocellular carcinoma (HCC) through the YAP/SLC7A11 signaling pathway 20. TLR2, a key player in innate immunity, activates inflammatory and immune responses upon detecting pathogen-associated molecular patterns and damage-associated molecular patterns. Our findings highlight that GATA6 responds to inflammatory signals via the TLR2 pathway, altering AKR1C3 expression and thereby influencing NPC ferroptosis and IVDD progression. These findings provide valuable insights into developing novel targeted treatments for IVDD.

## Materials and Methods

### Patients and IVDD tissue samples

Clinical and transcriptomic data related to IVDD were retrieved for bioinformatic analysis from the Gene Expression Omnibus (GEO) database, specifically the GSE70362 dataset. Information on ferroptosis-related genes was obtained from the FerrDb V2 database. For validation, IVD tissue samples were collected from patients diagnosed with IVDD who had received treatment at Southern Hospital. Patients with a history of prior lumbar surgery were excluded from the study. The disc samples obtained during surgical procedures. The degeneration grades of the IVD were evaluated using the Pfirrmann grading system, a widely accepted classification method for assessing IVDD based on MRI signal intensity, disc structure, and disc height 21. All tissue samples were promptly frozen in liquid nitrogen and stored for subsequent analysis. Quantitative real-time polymerase chain reaction (RT-PCR) was employed to assess the expression levels of the target genes.

The study protocol was reviewed and approved by the Ethical Review Committee (NFEC-202205-K18), and written informed consent was obtained from all participants, in compliance with the principles outlined in the Declaration of Helsinki.

### RNA extraction and quantitative RT-PCR

Total RNA was isolated from IVDD cell lines and freshly harvested tissue samples using TRIzol Reagent (ThermoFisher Scientific), in accordance with the manufacturer's protocol. Subsequently, 2μg of RNA was reverse-transcribed into complementary DNA using Oligo(dT) primers (Life Technologies) and RevertAid Reverse Transcriptase (ThermoFisher Scientific). Gene expression levels were quantified using the comparative cycle threshold (Ct) method with SuperReal PreMix SYBR Green (TIANGEN, FP204-02) on an Applied Biosystems 7500 Fast RT-PCR system (Life Technologies). Primer sequences are provided in **Supplementary Table 1**.

### Isolation and culture of NPCs

NPCs were isolated and cultured according to the following procedure: NP tissues were enzymatically digested with 0.2% type II collagenase (Solarbio) for 4 hours at 37°C. Following digestion, the tissues were washed with phosphate-buffered saline (PBS) and transferred to DMEM/F12 medium (ThermoFisher Scientific), supplemented with 10% fetal bovine serum (FBS, Gibco, USA) and 1% penicillin/streptomycin (Gibco). Cells were cultured in a humidified incubator at 37°C with 5% CO2. Upon reaching confluency, cells were passaged using 0.25% Trypsin-EDTA (Gibco). Cells from the second passage were used for subsequent experiments.

### Knockdown and overexpression in NPCs

NPCs were seeded at a density of 2×10^5^ cells per well in six-well plates 24 hours prior to transfection. To enable gene knockdown, cells were maintained in 2.5 mL of antibiotic-free growth medium per well. Transfections were carried out using Lipofectamine™ 2000 (ThermoFisher Scientific) in serum- and antibiotic-free OptiMem™ I Reduced Serum medium (ThermoFisher Scientific), following the manufacturer's instructions. Plasmids containing short hairpin RNA (shRNA) specific for human GATA6, AKR1C3, and TLR2, or scrambled shRNA as a control (ThermoFisher Scientific), were used for knockdown. For overexpression studies, plasmids encoding GATA6, AKR1C3, and TLR2 were employed. Cells were harvested for Western blot analysis at 24-, 48-, and 72-hours post-transfection. The corresponding plasmid sequences are listed in **Supplementary Table 2**.

### Western blotting

Total protein extraction from tissues or cells was performed using ice-cold radioimmunoprecipitation assay (RIPA) buffer supplemented with protease and phosphatase inhibitors (Cell Signaling Technology). Proteins were separated by sodium dodecyl sulfate-polyacrylamide gel electrophoresis (SDS-PAGE), and subsequently transferred to polyvinylidene fluoride (PVDF) membranes (Merck Millipore). The membranes were blocked for 1 hour at room temperature using 5% skim milk in Tris-buffered saline containing Tween-20 (TBST). They were then incubated overnight at 4°C with primary antibodies, followed by treatment with horseradish peroxidase (HRP)-conjugated secondary antibodies for 1 hour at room temperature. Protein visualization was conducted using enhanced chemiluminescence detection kits (Thermo Scientific), and the membranes were imaged using the ChemiDoc XRS+ System (Bio-Rad) in combination with Immobilon Western Chemiluminescent HRP Substrate (Millipore). Full and uncropped images of the Western blot results are provided in **Supplementary Table 3**.

### Cell counting kit-8 (CCK-8) assay

To assess cell viability, cells were seeded at a density of 1×10^4 cells per well in 48-well plates. At the designated time points, 30 μL of CCK-8 solution (Dojindo) was added to each well, followed by incubation for 1.5 hours. The optical density at 450 nm was then measured to quantify cell viability.

### MDA assay

The relative concentration of malondialdehyde (MDA) in cellular or tumor lysates was quantified using the Lipid Peroxidation (MDA) Assay Kit (ab118970, Abcam) following the manufacturer's protocol. The MDA adduct was detected colorimetrically by measuring the optical density at 532 nm.

### 4-HNE assay

The levels of 4-hydroxynonenal (4-HNE) were quantified using an enzyme-linked immunosorbent assay (ELISA) kit (MBS161454, MyBioSource), according to the manufacturer's instructions.

### Lipid ROS assay

To evaluate lipid ROS levels, cells were seeded in 96-well plates at a density of 1 × 10⁴ cells per well. A working solution of lipid ROS detection reagent (R252, Dongren) was added to the culture medium (200 μL per well), and cells were incubated at 37 °C with 5% CO₂ for 30 minutes. Lipid ROS levels were then analyzed using a fluorescent enzyme labeling instrument, with fluorescence intensity measured at an excitation/emission wavelength of 520 nm.

### FerroOrange detection kit

The FerroOrange detection kit (Dojindo) was used to quantify labile ferrous ions (Fe^2+^) in cells. Cells were added to 6-well plates and subjected to the respective treatments. After treatment, the culture medium was removed and the cells were washed twice with phosphate-buffered saline (PBS). The cells were then incubated in 1 μM FerroOrange solution in DMEM without FBS for 30 minutes at 37°C. After incubation, cells were washed three times with PBS to remove unbound probe. Fluorescence images were captured using a fluorescence microscope equipped with appropriate filters, and the fluorescence intensity was quantified using ImageJ software. The excitation/emission wavelengths for FerroOrange were set to 542/572 nm.

### Establishment of a murine IVDD model

A murine model of IVDD was developed for *in vivo* validation. C57BL/6 male mice (4 per group) underwent surgical intervention under general anesthesia. A posterolateral approach to the lumbar spine was used, and the L4/L5 intervertebral disc was punctured using either a 35-gauge or 33-gauge needle. The disc was anatomically subdivided into central, ventral, and dorsal regions, with the needle being described as traversing the “central region” if it passed through the central area, or the “ventral” or “dorsal region” based on its partial infiltration into those zones. Mice were euthanized 12 weeks after surgery, and degenerative changes in the punctured discs were qualitatively assessed under light microscopy using hematoxylin and eosin (H&E) and Safranin O/Fast Green staining.

### Statistical analysis

Statistical analyses were performed using SPSS version 22.0 (SPSS Inc.), Prism 7.0 (GraphPad Software), and R version 4.2.3. Data were presented as the mean ± standard deviation, based on a minimum of three independent experiments. For quantitative data, comparisons were made using a two-tailed Student's t-test or the Wilcoxon matched-pairs signed-rank test, where appropriate. For categorical data, the Fisher's exact test was used. Prognostic factors were assessed using univariate and multivariate Cox proportional hazards regression analyses. Bioinformatics analysis was primarily conducted within the R environment, employing established R packages and algorithms. Statistical significance was defined as a two-tailed p-value < 0.05.

## Results

### Construction of a ferroptosis-related gene risk coefficient model in IVDD

**Figure 1:**
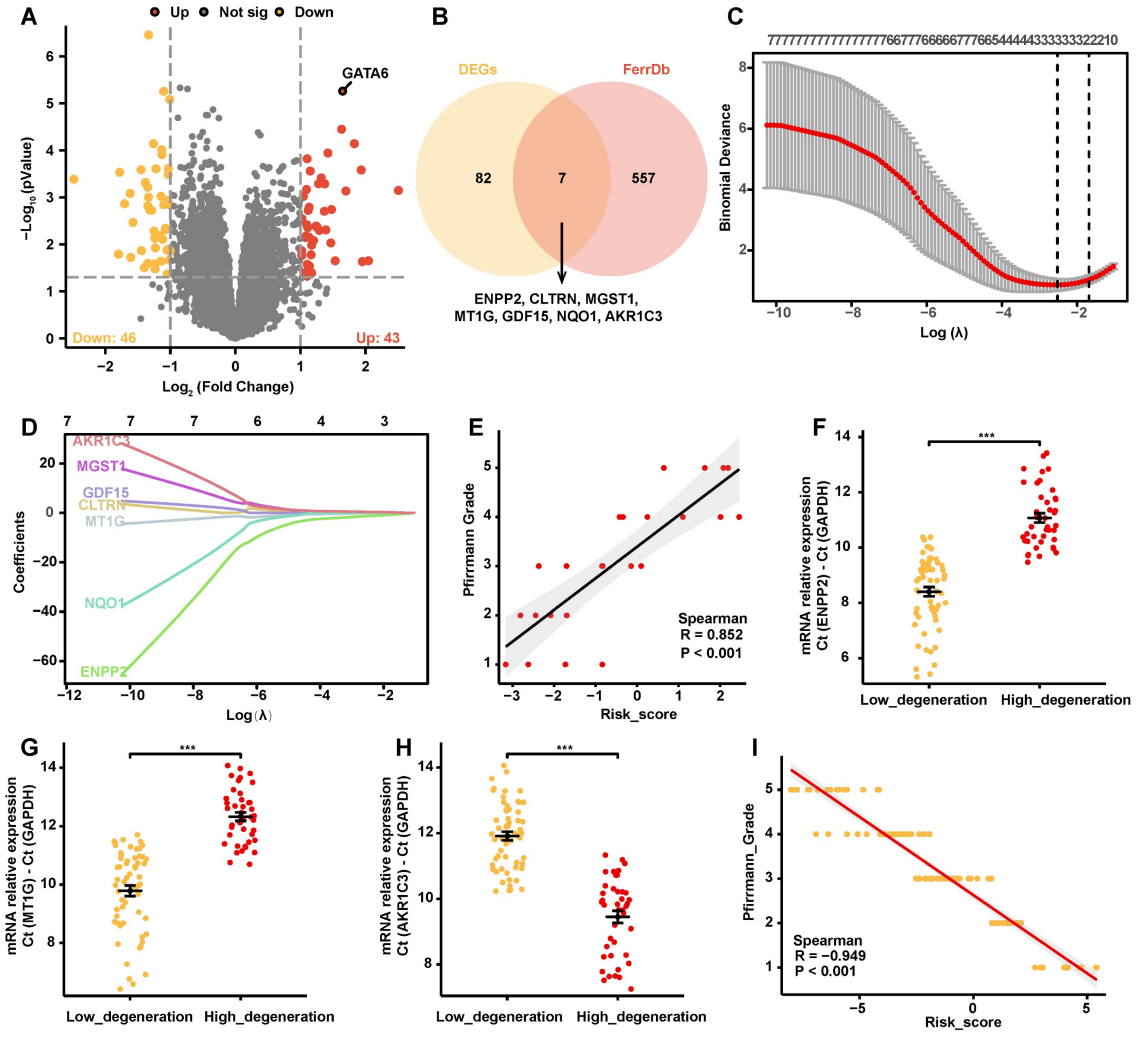
** Construction of a ferroptosis-related gene risk model.** A. Volcano plot depicting differentially expressed genes (DEGs) between degeneration grades 1-3 and 4-5 in the GSE70362 dataset (abs(logFC) > 1, p < 0.05). B. Venn diagram showing the intersection of DEGs with ferroptosis-related genes from the FerrDb V2 database, resulting in seven genes (ENPP2, CLTRN, MGST1, MT1G, GDF15, NQO1, AKR1C3). C-D. LASSO regression analysis identifying three non-zero parameter genes (ENPP2, MT1G, and AKR1C3). (C) Lasso coefficient selection plot. (D) Diagnostic Lasso variable trajectory plot. E. Correlation analysis scatter plot of risk score with Pfirrmann degeneration grade in the GSE70362 dataset. F-H. Changes in mRNA levels of ENPP2 (F), MT1G (G), and AKR1C3 (H) in low- versus high-grade degeneration from 100 clinical specimens. I. Correlation scatter plots of ENPP2, MT1G, and AKR1C3 mRNA levels with risk score in 100 clinical disc tissue samples. *p < 0.05, **p < 0.01, ***p < 0.001.

To assess the impact of ferroptosis on the progression of IVDD, we conducted a comprehensive differential gene expression (DEG) analysis using the GSE70362 dataset. Patient-derived samples were categorized by Pfirrmann degeneration grades into mild (grades 1-3) and severe (grades 4-5), and 43 upregulated and 46 downregulated genes were identified in the severe group (**Figure 1A, Supplementary Table 4**). A list of 844 ferroptosis-associated genes, including drivers, inhibitors, markers, and genes with undetermined functions were retrieved from the FerrDb V2 database (**Supplementary Table 5**). Intersecting these genes with the identified DEGs yielded seven overlapping genes: ENPP2, CLTRN, MGST1, MT1G, GDF15, NQO1, and AKR1C3 (**Figure 1B**). These overlapping genes were examined using Employing Least Absolute Shrinkage and Selection Operator (LASSO) cox regression analysis with ten-fold cross-validation via “glmnet” package of R, and a predictive risk coefficient model focused on three key genes with significant non-zero coefficients was developed: ENPP2, MT1G, and AKR1C3 (**Figure 1C, 1D, Supplementary Table 6**).

The model's accuracy was confirmed through a significant positive correlation between the risk scores generated from the GSE70362 dataset and Pfirrmann degeneration grades (**Figure 1E**). Further validation was achieved by quantifying the mRNA levels of ENPP2, MT1G, and AKR1C3 in disc tissue specimens from 100 clinical cases. Notably, ENPP2 and MT1G levels were significantly reduced in the high-grade degeneration group, while AKR1C3 expression was markedly increased (**Figure 1F-H**). Corresponding risk scores were then calculated, revealing a strong positive correlation with Pfirrmann grades, thereby reinforcing the model's predictive efficacy (**Figure 1I**).

Taken together, these results indicate that the ferroptosis-related gene risk coefficient model effectively predicts the severity of IVDD.

### Positive correlation of GATA6 and AKR1C3 with IVDD development

**Figure 2:**
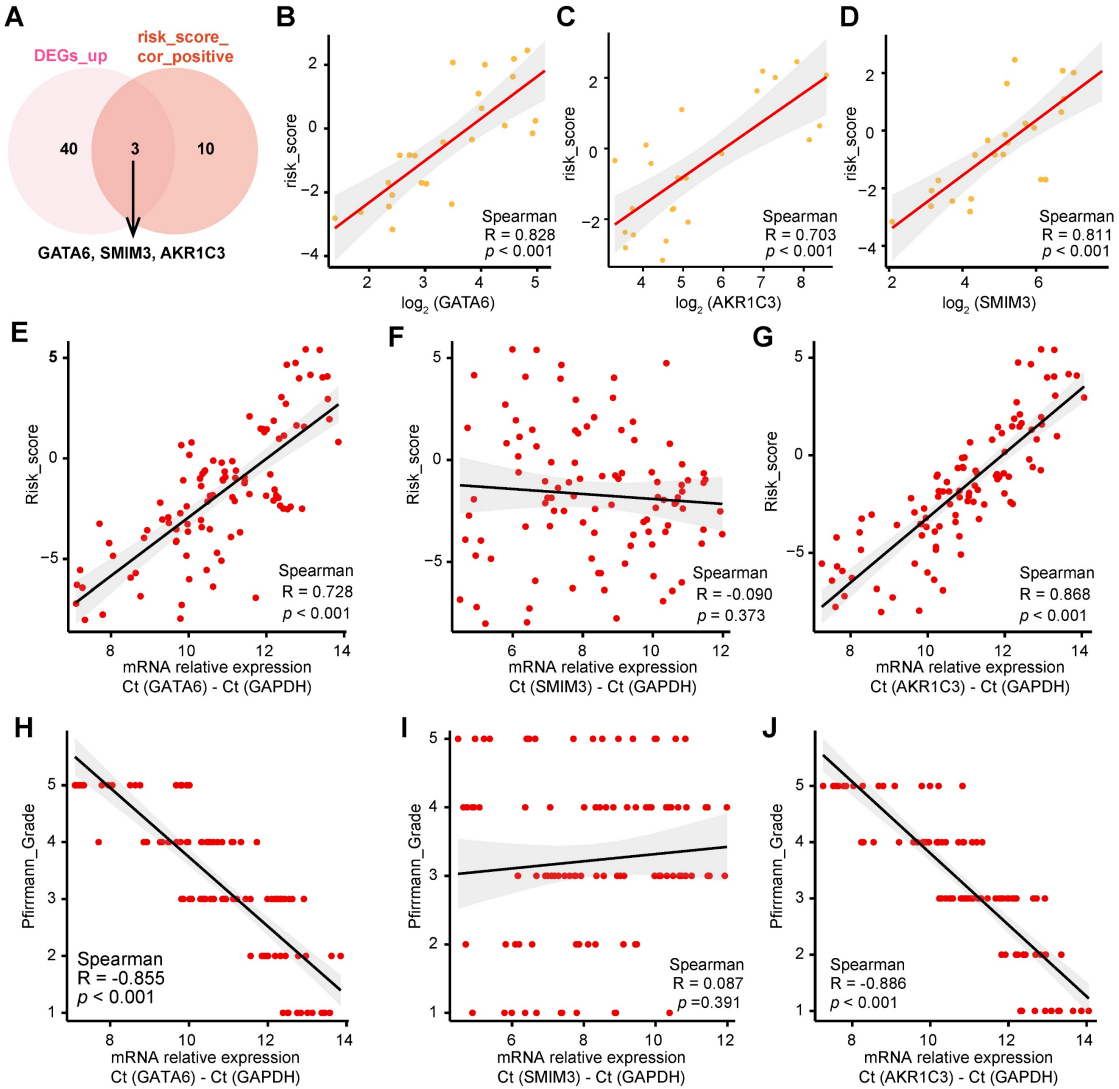
** Positive correlation of GATA6 and AKR1C3 with IVDD development.** A. Venn diagram showing the intersection of upregulated DEGs from Figure 1A with genes positively correlated with risk scores in the GSE70362 dataset. B-D. Correlation between GATA6 (B), AKR1C3 (C) and SMIM3 (D) mRNA levels and risk scores in 100 clinical IVD samples. E-G. Correlation between GATA6 (E), AKR1C3 (F) and SMIM3 (G) mRNA levels and risk scores in 100 clinical specimens. H-J. Correlation between GATA6 (H), AKR1C3 (I) and SMIM3 (J) mRNA levels and Pfirrmann degeneration grade in 100 clinical specimens.

To determine the molecular mechanisms underlying ferroptosis in IVDD, we performed a correlation analysis between gene expression profiles and the corresponding risk scores derived from the GSE70362 dataset, applying a significance threshold of |cor| > 0.75 and p < 0.05 (**Supplementary Table 7**). By comparing these data with the upregulated DEGs in Figure 1A, we identified three genes—GATA6, SMIM3, and AKR1C3—that were strongly correlated with risk scores (**Figure 2A-D**). Validation studies conducted on 100 clinical cases confirmed a significant positive correlation between mRNA levels of GATA6 and AKR1C3 and both risk scores and Pfirrmann grades, whereas SMIM3 did not show a significant correlation (**Figure 2E-J**). Among these, GATA6 displayed the most pronounced variation, warranting further investigation.

### GATA6 promotes ferroptosis in NPCs and contributes to IVDD progression

**Figure 3:**
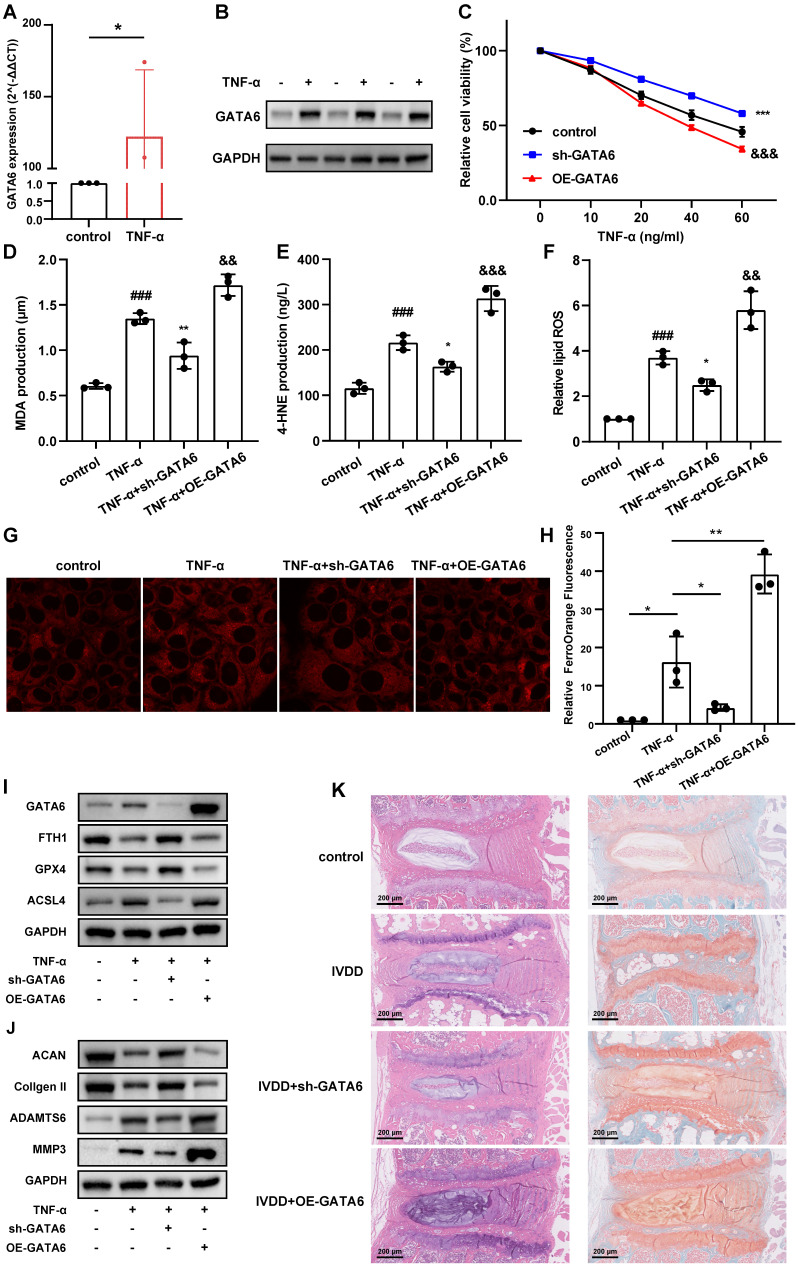
** GATA6 enhances ferroptosis and facilitates IVDD development.** A-B. Comparative analysis of GATA6 mRNA (A) and protein (B) expression levels in control versus TNF-α-treated NPCs. C: Relative cell viability of corresponding treated NPCs assessed by the CCK-8 assay. D-F. MDA (D), 4-HNE (E), and lipid ROS (F) measurements in NPCs following indicated treatment. G-H. Evaluation of intracellular ferrous ion concentrations in NPCs treated with sh-GATA6 or OE-GATA6. G: Fluorescence imaging. H: Quantitative analysis. sh-GATA6: GATA6 shRNA. OE-GATA6: GATA6 overexpression. I-J. Western blot analysis of ferroptosis-related proteins (GPX4, FTH1, ACSL4) (I) and ECM degradation markers (ACAN, Collagen II, ADAMTS6, MMP3) (J) in different treated NPCs. K. H&E and Safranin O/Fast Green staining assessments of disc degeneration in a bipedal standing mouse model following different treatment. Experimental group compared to control group: ##p < 0.01, ###p < 0.001. sh-GATA6 group compared to Experimental group: *p < 0.05, **p < 0.01, ***p < 0.001. OE-GATA6 group compared to Experimental group: &p < 0.05, &&p < 0.01, &&&p < 0.001.

To validate this hypothesis, we treated NPCs with tumor necrosis factor (TNF)-α to establish a model of NPC degeneration. GATA6 mRNA and protein levels were markedly elevated in degenerating NPCs compared to the control group (**Figure 3A, 3B**). Silencing GATA6 significantly enhanced cellular activity, while its overexpression suppressed activity, as measured by the CCK-8 assay (**Figure 3C**). To determine if GATA6 promotes cell death via ferroptosis, MDA, 4-HNE, lipid ROS and the FerroOrange detection kit were performed. GATA6 knockdown significantly reduced cellular ferrous ion levels (**Figure 3D-H**). Furthermore, GATA6 inhibition attenuated ferroptosis, as evidenced by the upregulation of ferroptosis suppressors GPX4 and FTH1 and the downregulation of the ferroptosis inducer ACSL4 (**Figure 3I**). Conversely, GATA6 overexpression had the opposite effect on these levels of ferroptosis suppressors and inducer (**Figure 3I**). We also investigated the influence of GATA6 on ECM degradation, a key feature of IVDD. GATA6 knockdown significantly inhibited ECM degradation, as indicated by the increased expression of ECM components ACAN and collagen II, and reduced levels of ECM degradation markers ADAMTS6 and MMP3 (**Figure 3J**). Conversely, GATA6 overexpression had the opposite effect (**Figure 3J**). These findings supported the hypothesis that inhibiting GATA6 can slow the progression of IVDD. Additionally, as GATA6 has been implicated in the regulation of apoptosis across various tissues, we investigated its role in apoptosis in NPCs. Using TUNEL staining and apoptosis marker analysis, our results showed that GATA6 had no significant impact on apoptosis in NPCs (**Figure S1A-B**).

To corroborate these* in vitro* findings, we established an *in vivo* mouse model of IVDD using annulus fibrosus needle puncture. Mice were treated with either saline (control) or an adeno-associated virus engineered to knock down or overexpress GATA6 22. The IVDD group showed a reduced population of NPCs, diminished ECM, thinning of the annulus fibrosus, and disordered fiber arrangement (**Figure 3K**). These pathological features were exacerbated in the GATA6 overexpression group (**Figure 3K**). Conversely, the sh-GATA6 treated group showed improvement in these pathological markers, indicating that GATA6 knockdown mitigates IVDD progression *in vivo* (**Figure 3K**).

In summary, these findings suggest that GATA6 facilitates ferroptosis and ECM degradation in NPCs, thereby promoting the progression of IVDD.

### GATA6 promotes ferroptosis and degeneration in NPCs via AKR1C3

**Figure 4:**
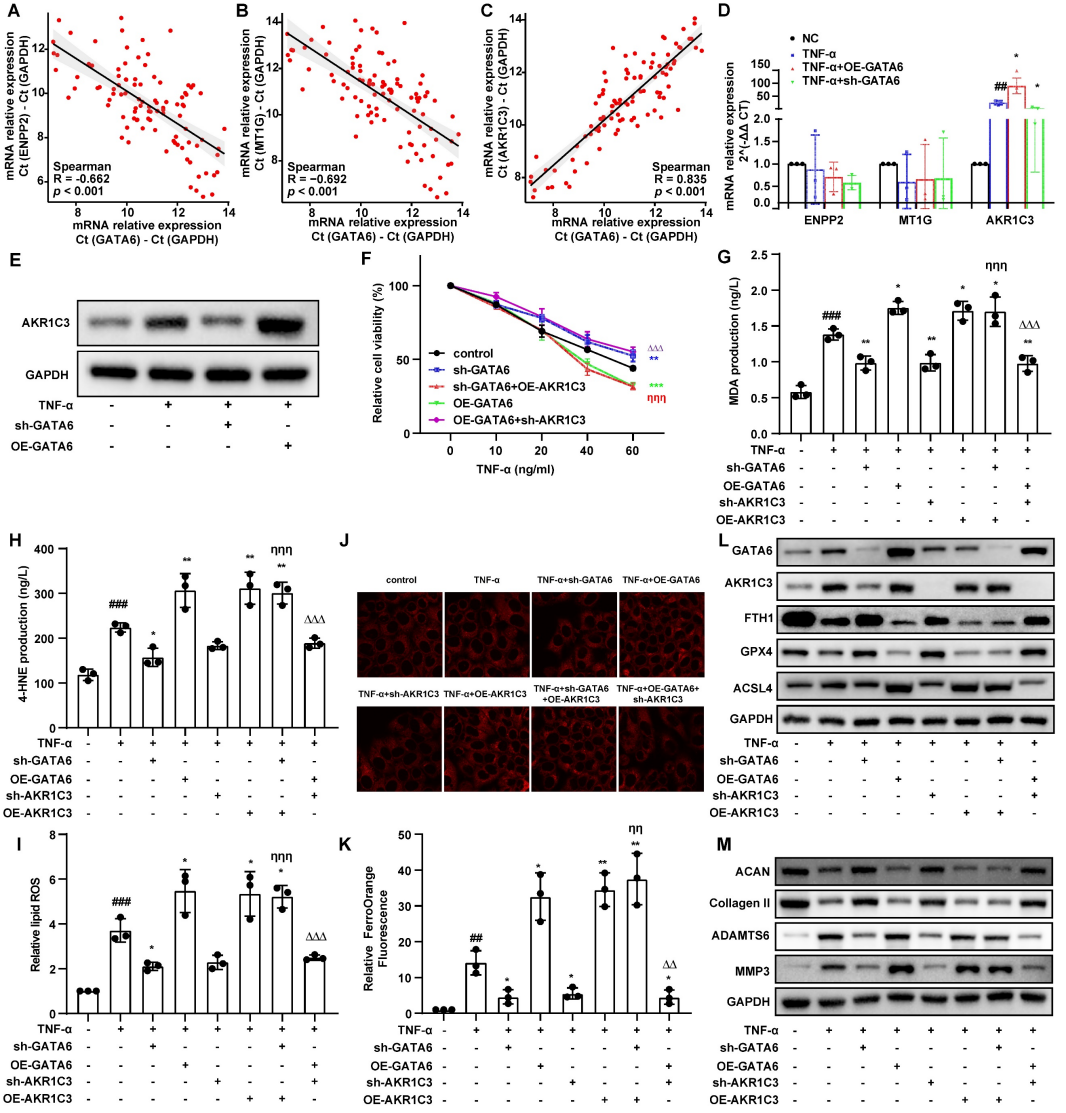
** GATA6 promotes ferroptosis and degeneration in NPCs through AKR1C3.** A-C. Correlation analysis scatter plots between GATA6 and AKR1C3 mRNA levels in clinical IVDD samples. D-E. Quantitative analysis of AKR1C3 mRNA (D) and protein (E) expression levels in NPCs subjected to GATA6 knockdown or overexpression. F. Viability of NPCs following respective treatments evaluated using the CCK-8 assay. G-I. MDA (G), 4-HNE (H), and lipid ROS (I) measurements in NPCs following indicated treatment. J-K. Fluorescence imaging (J) coupled with statistical analysis (K) illustrating the modulation of intracellular ferrous ion concentrations by GATA6 and AKR1C3 changes. L-M. Western blot analysis of ferroptosis markers (GPX4, FTH1, ACSL4) and ECM degradation markers (ACAN, Collagen II, ADAMTS6, MMP3) in NPCs subjected to corresponding treatments. Experimental group compared to control group: ##p < 0.01, ###p < 0.001. Indicated group compared to TNF-α group: *p < 0.05, **p < 0.01, ***p < 0.001. Experimental group compared to sh-AKR1C3 group: &&p < 0.01, &&&p < 0.001. sh-GATA6 group compared to sh-GATA6+OE-AKR1C3 group: ηη p<0.01, ηηη p<0.001. OE-GATA6 group compared to OE-GATA6+sh-AKR1C3 group: ΔΔ p<0.01, ΔΔΔ p<0.001.

Building on our findings, we investigated the role of GATA6 in regulating ferroptosis in NPCs. Given the observed correlation between IVDD severity and ferroptosis-related genes ENPP2, MT1G, and AKR1C3 (**Figure 1C, 1D**), we hypothesized that GATA6 may regulate these genes to activate the ferroptosis pathway. Correlation analysis of clinical IVDD samples demonstrated a positive association between GATA6 and AKR1C3 mRNA levels, along with a negative correlation between GATA6 and ENPP2/MT1G, suggesting a potential regulatory interaction (**Figure 4A-C**). Quantitative RT-PCR revealed that GATA6 knockdown significantly reduced AKR1C3 expression, while GATA6 overexpression increased its expression (**Figure 4D**). Western blot analysis confirmed these findings, showing modulation of AKR1C3 protein levels by GATA6 (**Figure 4E**). CCK-8 assay demonstrated that AKR1C3 overexpression reversed the enhanced viability caused by GATA6 knockdown, while AKR1C3 knockdown mitigated the inhibitory effect of GATA6 overexpression on cell viability (**Figure 4F**). Contrary to prior studies suggesting that AKR1C3 inhibits ferroptosis in cancer cells 20, our results revealed that AKR1C3 suppression significantly reduced cell death and intracellular ferrous ion levels in NPCs, while its upregulation had the opposite effect, highlighting the role of AKR1C3 in promoting NPC ferroptosis (**Figure 4G-K**). Western blot analysis further supported these results, showing that alterations in ferroptosis markers (GPX4, FTH1, ACSL4) and ECM components (ACAN, collagen II, ADAMTS6, MMP3) due to GATA6 inhibition could be reversed by AKR1C3 re-expression. Conversely, AKR1C3 knockdown counteracted the effects of GATA6 overexpression (**Figure 4L-M**). Together, these findings suggest that GATA6 drives NPC ferroptosis and degeneration by modulating AKR1C3.

### GATA6 promotes AKR1C3 expression via the TLR2-mediated pathway

**Figure 5:**
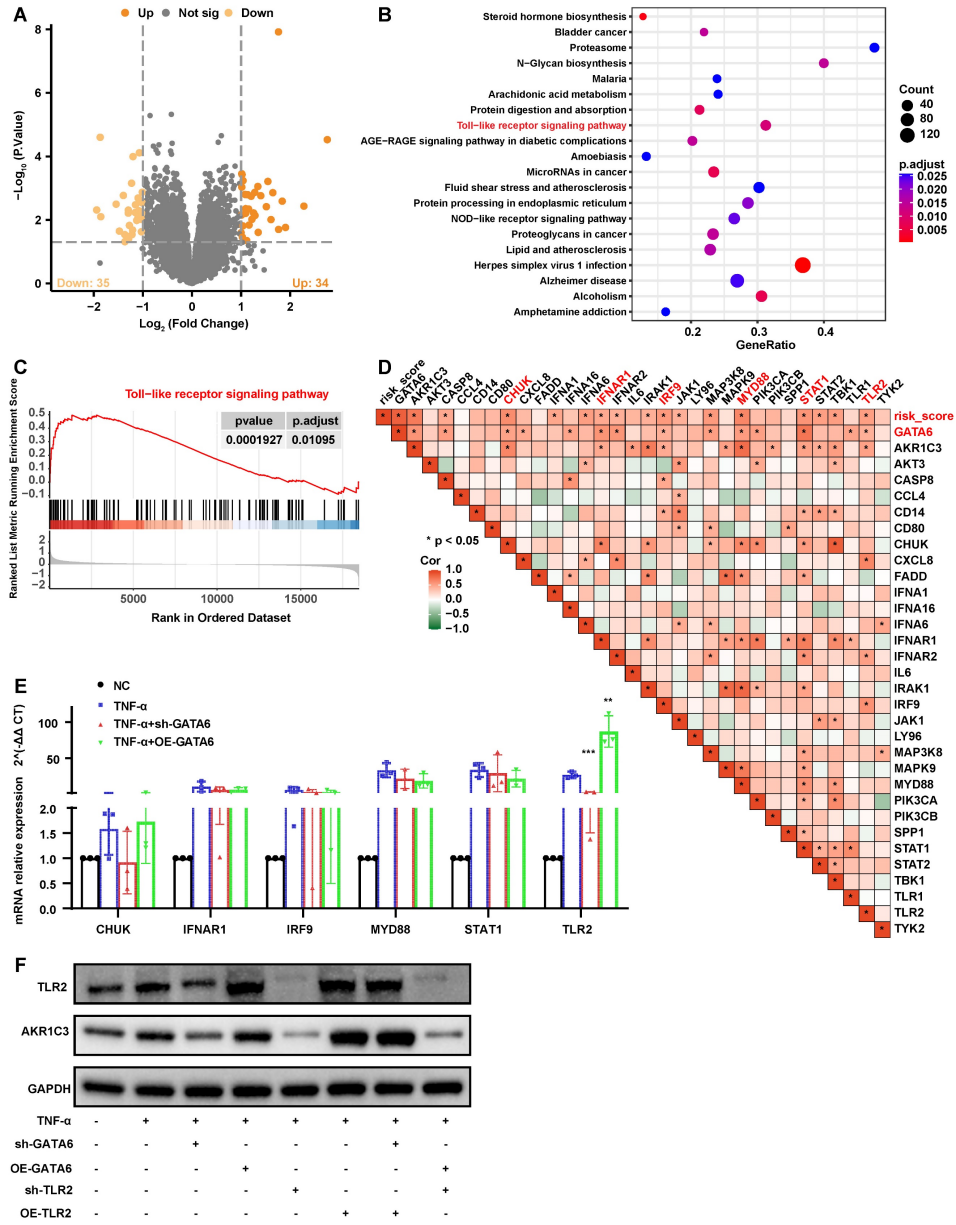
** GATA6 modulates AKR1C3 expression via TLR2 in the progression of IVDD.** A. Volcano plot depicting differentially expressed genes between low and high GATA6 expression groups in the GSE7362 dataset (|logFC| > 1 and p < 0.05). B. GSEA analysis of the DEGs from graph A, sorted by enrichment Score, displaying the top 20 signaling pathways. C. GSEA plot of the Toll-like receptor signaling pathway, which is significantly upregulated in the high GATA6 expression group. D. Heatmap correlating genes enriched in the Toll-like receptor signaling pathway with GATA6 expression levels and risk score. E. Quantitative RT-PCR analysis of mRNA levels for CHUK, IFNAR1, IRF9, MYD88, STAT1, and TLR2 in NPCs subjected to GATA6 and TLR2 knockdown or overexpression. F. Western blot analysis of AKR1C3 and TLR2 protein levels in NPCs with GATA6 and TLR2 knockdown or overexpression. Indicated group compared to Experimental group: **p < 0.01, ***p < 0.001.

To further elucidate how GATA6 facilitates ferroptosis and degeneration in NPCs through the regulation of AKR1C3, we analyzed the GSE7362 dataset. Samples were stratified by GATA6 expression levels, leading to the identification of 35 upregulated and 34 downregulated genes using a threshold of |logFC| > 1 and p < 0.05 (**Figure 5A, Supplementary Table 8**). Gene Set Enrichment Analysis (GSEA) revealed a significant upregulation of the TLR signaling pathway in the high GATA6 expression group (**Figure 5B, 5C, Supplementary Table 9**), highlighting the critical role of this pathway in GATA6-mediated NPC ferroptosis and IVDD pathology.

Correlation heatmaps were employed to examine the association between key genes in the TLR pathway, GATA6, risk scores, and AKR1C3 expression. Six genes, CHUK, IFNAR1, IRF9, MYD88, STAT1, and TLR2 were significantly associated with GATA6, risk scores, and AKR1C3 (**Figure 5D**). Quantitative RT-PCR confirmed that TLR2 mRNA levels decreased following GATA6 knockdown and increased with GATA6 overexpression (**Figure 5E**). Western blot analysis further demonstrated that TLR2 overexpression reversed the reduction in AKR1C3 expression caused by GATA6 knockdown, while TLR2 knockdown reversed the increase in AKR1C3 expression induced by GATA6 overexpression (**Figure 5F**). These results indicate that GATA6 enhances AKR1C3 expression in NPCs via the TLR2-mediated pathway.

### GATA6 drives ferroptosis and degeneration in NPCs through TLR2

**Figure 6:**
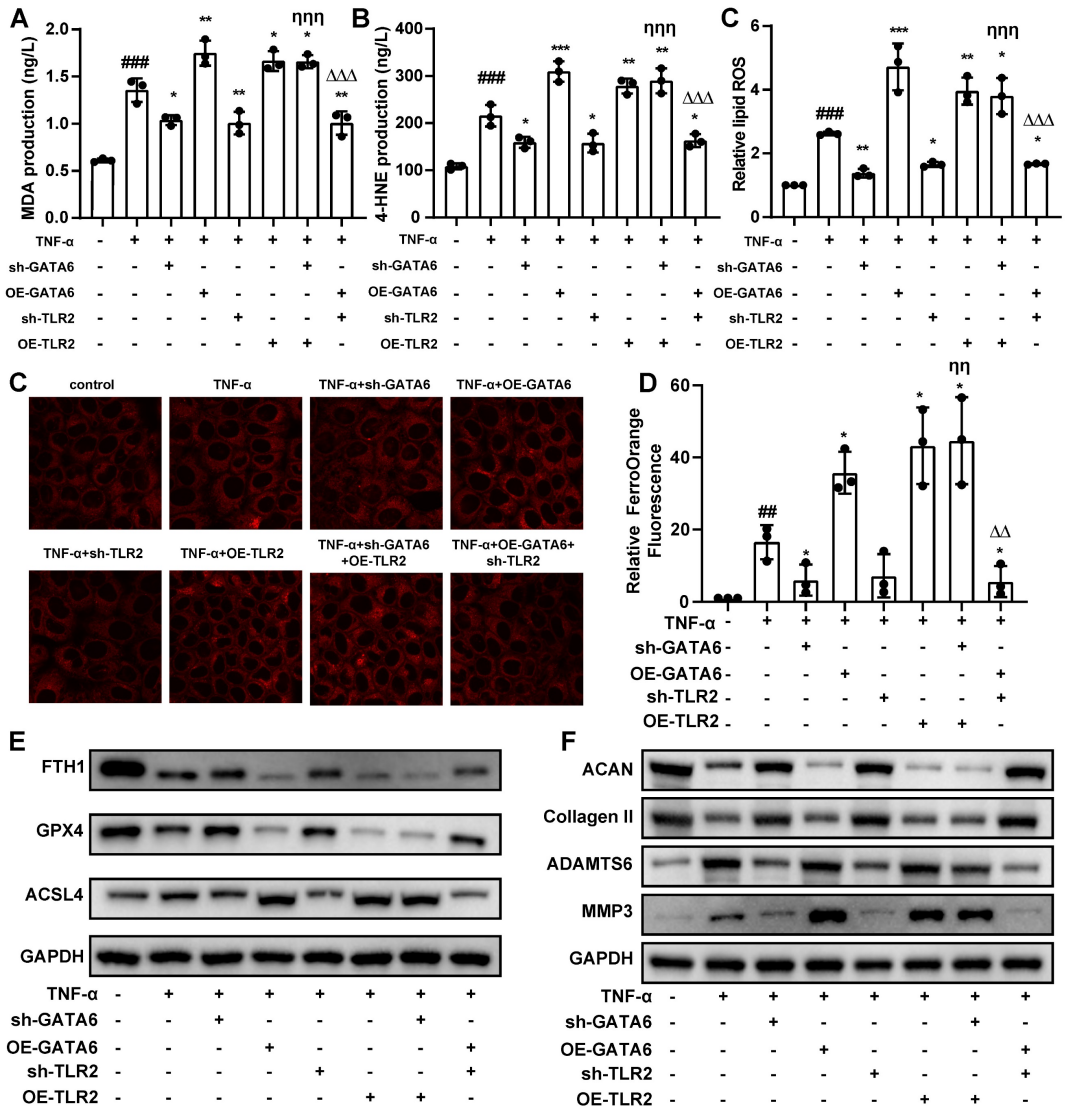
** GATA6 regulates ferroptosis and degeneration in NPCs via TLR2.** A-C. MDA (A), 4-HNE (B), and lipid ROS (C) measurements in NPCs following indicated treatment. D-E. Ferro Orange assay quantifying ferrous ion levels in NPCs under the respective treatment conditions. D: Fluorescence images. E: Statistical analysis. F-G. Western blot analysis of ferroptosis markers (F) and ECM degeneration markers (G) in the corresponding treatment groups. Experimental group compared to control group: ##p < 0.01, ##p < 0.001. Indicated group compared to experimental group: *p < 0.05, **p < 0.01, ***p < 0.001. sh-GATA6 group compared to sh-GATA6+OE-TLR2 group: ηη p<0.01, ηηη p<0.001. OE-GATA6 group compared to OE-GATA6+sh-TLR2 group: ΔΔ p<0.01, ΔΔΔ p<0.001.

These findings suggest a pivotal role for TLR2 in mediating the pathological effects of GATA6 on NPCs. Supporting this hypothesis, TLR2 knockdown in NPCs led to a reduction in ferroptosis levels, while its overexpression exacerbated these effects (**Figure 6A-D**). Furthermore, overexpression of TLR2 in a GATA6 knockdown model mitigated the reduction in ferroptosis caused by GATA6 suppression. Conversely, simultaneous knockdown of TLR2 and overexpression of GATA6 attenuated GATA6-induced ferroptosis and ferrous ion release (**Figure 6A-D**). Western blot analysis of ferroptosis and degeneration markers corroborated the role of TLR2 as a downstream effector in the GATA6-regulated degenerative pathway in NPCs (**Figure 6E, 6F**). Together, these results demonstrate that GATA6 can regulate NPC ferroptosis and degeneration through the TLR2 signaling pathway.

## Discussion

The results of this study provide important insights into the role of ferroptosis in the pathophysiology of IVDD, a condition with substantial global health implications due to its association with disability. Ferroptosis, a regulated form of cell death characterized by iron dependency and lipid ROS accumulation, has been identified as a critical contributor to IVDD progression. Prior research has emphasized the therapeutic potential of inhibiting ferroptosis to alleviate IVDD, as demonstrated by the worsening of disc cell degeneration and lipid peroxidation following oxidative stress induced by tert-butyl hydroperoxide. In line with this, we developed a risk coefficient model that showed a strong positive correlation between risk scores and Pfirrmann degeneration grades, underscoring its diagnostic accuracy. This model was further validated with clinical samples, confirming a correlation between risk scores and IVDD severity.

Further examination of the regulatory mechanisms showed that GATA6 and AKR1C3 are key factors strongly correlated with ferroptosis risk scores, with GATA6 showing the most significant variation. GATA6 is a critical transcription factor, widely expressed in various cell types and involved in processes such as differentiation and survival 23, 24. Previous research in non-small cell lung cancer has demonstrated that GATA6 downregulation promotes cell migration, proliferation, and cell cycle progression 15, while its deletion in chronic inflammation reduces monocyte recruitment and pro-inflammatory macrophage formation, thus attenuating atherosclerosis 17. Furthermore, GATA6 has been implicated in vascular smooth muscle cell senescence-related arterial calcification by counteracting anti-aging factors such as SIRT6 and impairing DNA damage repair 16. In our study, GATA6 was found to play a similar detrimental role in IVDD by accelerating ferroptosis in NPCs, thereby exacerbating IVDD progression. Notably, GATA6 knockdown mitigated ECM degradation *in vitro* and slowed IVDD progression *in vivo*, in contrast to its protective role observed in cerebral ischemia-reperfusion injury, likely due to differences in vascular supply between the brain and NPCs 25.

Further investigation revealed that GATA6 promotes ferroptosis by regulating AKR1C3 expression, a process mediated by TLR2. AKR1C3 is a member of the aldo-ketoreductase family, catalyzes key redox reactions integral to biosynthesis, metabolism, detoxification, and redox balance 26. In asthma, AKR1C3 overexpression has been shown to attenuate ferroptosis markers, while its silencing enhances them 27. Similarly, AKR1C3 has been associated with protection against ferroptosis in HCC. However, our study presents a contrasting scenario where AKR1C3 overexpression promotes ferroptosis in NPCs, while its downregulation reduces it. Given AKR1C3's role in promoting pro-inflammatory factors via NF-κB activation in HCC 28, and its association with local inflammatory mediators in rheumatoid arthritis 29, this discrepancy may reflect the unique hypoxic and inflammatory environment of IVD, which could alter AKR1C3's functional dynamics. Future studies should investigate the specific regulatory mechanisms of AKR1C3 in ferroptosis, including its interaction with oxidative stress and inflammatory pathways, to better understand its tissue-specific roles and potential as a therapeutic target in IVDD.

TLR signaling has been implicated in the inflammatory responses linked to IVDD and disc metabolic alterations 9. Activation of TLRs can induce disc degeneration and stimulate the release of pro-inflammatory cytokines such as interleukin (IL)-6, TNF-α and interferon-γ 30. Chronic stimulation of TLRs by ECM fragments from degenerated discs can further facilitate bone marrow-to-fat conversion, as observed in Modic changes 31. Notably, silencing TLR2 has been shown to reduce *Propionibacterium acnes*-induced apoptosis in NPCs 32. TLR2 has also been reported to play a crucial role in ferroptosis, with TLR2 agonists enhancing and antagonists inhibiting the phagocytic clearance of ferroptosis cells 33. Our findings further support the role of TLR2 in ferroptosis, demonstrating that TLR2 mediates the regulation of AKR1C3 by GATA6 in NPC ferroptosis and IVDD. Moreover, downregulation of GATA6 has been shown to reduce inflammation, infiltration, and mucus production by inhibiting TLR2 signaling in asthma models 34.

The risk coefficient model and the GATA6-TLR2-AKR1C3 axis hold significant potential for clinical applications. The model could serve as an early diagnostic tool for IVDD, enabling the prediction of disease progression based on GATA6 and AKR1C3 expression levels. Moreover, therapeutic strategies targeting the GATA6-TLR2-AKR1C3 axis, such as small molecule inhibitors or modulation of the TLR2 pathway, present promising avenues for IVDD treatment. However, further clinical trials are necessary to validate the efficacy and safety of these strategies.

In conclusion, our study identified the critical role of ferroptosis in IVDD progression and established a risk coefficient model for assessing the disease. Additionally, the identification of the GATA6-TLR2-AKR1C3 axis as a key regulatory pathway offers insights into potential therapeutic targets for IVDD. Future research should focus on the regulatory mechanisms of this axis across different stages of IVDD to develop more effective treatments.

## Supplementary Material

Supplementary figure and tables.

## Abbreviations

NP: nucleus pulposus
IVDD: Intervertebral disc degeneration
NPCs: NP cells
ROS: reactive oxygen species
ECM: extracellular matrix
GATA6: GATA-binding protein 6
AKR1C3: Aldo-keto reductase family 1 member C3
TLR2: Toll-like receptor
HCC: hepatocellular carcinoma
CCK-8: Cell-Counting-Kit-8
H&E: Hematoxylin and Eosin
DEG: differential gene expression
LASSO: Least Absolute Shrinkage and Selection Operator
GSEA: Gene Set Enrichment Analysis
GEO: Gene Expression Omnibus
RT-PCR: Quantitative real-time PCR
PBS: phosphate-buffered saline
shRNA: short hairpin RNA
