# Supplementary material for: GATA6 Facilitates Progression of Intervertebral Disc Degeneration by Regulating Ferroptosis via Targeting TLR2/AKR1C3
Source: Int J Biol Sci. 2025 Jan 13;21(3):1174–86. doi: 10.7150/ijbs.102776 (PMC11781159; doi:10.7150/ijbs.102776)
Supplement: Supplementary file 1 — Supplementary figure and tables. [file ijbsv21p1174s1.pdf]

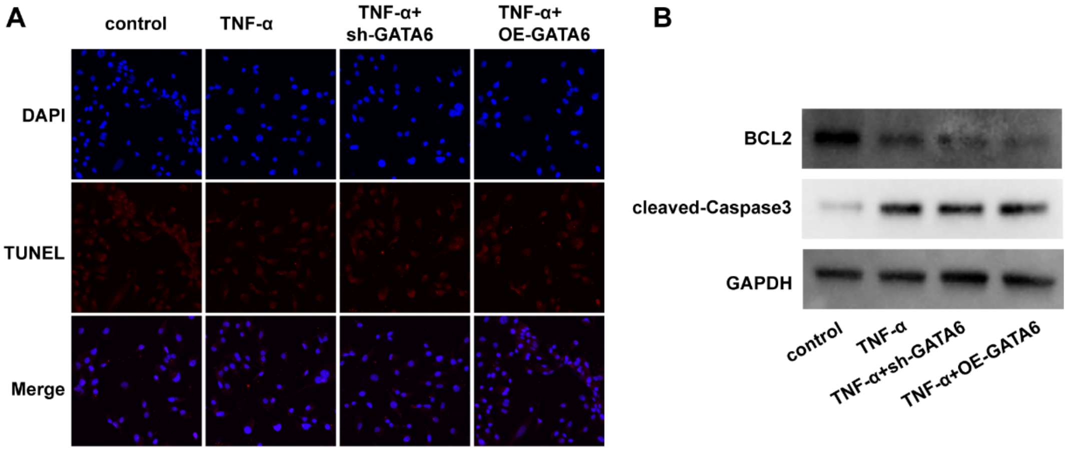

Figure 1: GATA6 has no significant impact on apoptosis in nucleus pulposus cells (NPCs).

A. Apoptosis levels were assessed using TUNEL staining in NPCs treated with sh-GATA6 or OE-GATA6.

B. Western blot analysis of apoptosis markers BCL2 and cleaved-caspase3 in NPCs treated with sh-GATA6 or OE-GATA6.

Supplementary Table 1 Primer sequences

Gene	primer sequences
Human GATA6 forward	CTCTACAGCAAGATGAACGG
Human GATA6 reverse	AAGGTGGTAGTTGTGGTGTG
Rattus GATA6 forward	CCAGCACAGACCTGTTGGAG
Rattus GATA6 reverse	GGCACAGAAATCACGCATCG
Human TLR2 forward	ATGTCACAGGACAGCACTGG
Human TLR2 reverse	CCAGGAATGAAGTCCCGCTT
Rattus TLR2 forward	GCCACAGGACTCAAGAGCAT
Rattus TLR2 reverse	GCCACAGGACTCAAGAGCAT
Human AKR1C3 forward	GGGTGTCAAACTTCAACCGC
Human AKR1C3 reverse	GTCTGATGCGCTGCTCATTG
Rattus AKR1C3 forward	CCATCGGGGTGTCCAACTTTA
Rattus AKR1C3 reverse	ACCGAAGATTTCTGTCCAGGTTA
Human ENPP2 forward	CGGCCCTGAGGAGAGTAGTT
Human ENPP2 reverse	TTGTCCTGACGAGTTTCCGC
Human MT1G forward	GAAGTCTTCCTAGGCCGTGATCT
Human MT1G reverse	AGCACTCACATTTCCGTCCA
Rattus CHUK forward	CTCAAGATGGCGAGACGTTA
Rattus CHUK reverse	TCGAGAACAGTGCACGAATGA
Rattus IFNAR1 forward	AGTGGATGTCCAAGGTGACAG
Rattus IFNAR1 reverse	AATGACGAAGCACCTCTCGG
Rattus IRF9 forward	TTATCACGGTGCGGATGGAG
Rattus IRF9 reverse	CAGACCAACGGTGTGGAGAA
Rattus MYD88 forward	CCTGTCTCCAGGTGTCCAAC
Rattus MYD88 reverse	ATCACGTTAGGGGCCTCTCT
Rattus STAT1 forward	ACAAAGACCATGCCTTCGGG
Rattus STAT1 reverse	ATTCGTGTTTATACTGCGCTCAT
Human GAPDH forward	GGAGCGAGATCCCTCCAAAAT
Human GAPDH reverse	GGCTGTTGTCATACTTCTCATGG
Rattus GAPDH forward	GCGAGATCCCGCTAACATCA
Rattus GAPDH reverse	ATTCGAGAGAAGGGAGGGCT

Supplementary Table 2 Targeting sequences of shRNA and overexpression

Gene	Targeting sequences
sh-GATA6	GGCAACGCATGCGGTCTCTACAGTAA

sh-TLR2	GGATCCGCTGTGATGGCAGCTCCAGGTTTCAAGAGA
sh-AKR1C3	GGATCCGCTGGAGAAGATCCTGAATAATTCAAGAGA
Rattus GATA6-F(piggybac)	AAGCTTATGGCCTTGACTGACGGCGG
Rattus GATA6-R(piggybac)	GAATTCTCAGGCCAGGCCAGAGCACACC
Rattus TLR2-F(piggybac)	GAATTCATGCTGCAAGCTCTTTGGCTCT
Rattus TLR2-R(piggybac)	CTCGAGTTAGGCCAGGCCAGAGGAGGAGAGG
Rattus AKR1C3-F(piggybac)	GAATTCATGAATTCCAAAATTCAGAAG
Rattus AKR1C3-R(piggybac)	CTCGAGTTAGTATTCAGATCGAGGAA

Supplementary Table 3 Antibodies information

Gene	Details
GATA6	Abcam, Cat# ab175349, UK
TLR2	Abcam, Cat# ab209217, UK
AKR1C3	Abcam, Cat# ab316864, UK
FTH1	Abcam, Cat# ab183781, UK
GPX	Abcam, Cat# ab125066, UK
ACSL4	Abcam, Cat# ab155282, UK
ACAN	Abcam, Cat# ab315486, UK
Coll II	Abcam, Cat# ab34712, UK
ADAMTS6	Abcam, Cat# ab50647, UK
MMP3	Abcam, Cat# ab52915, UK
GAPDH	Abcam, Cat# ab8245, UK
secondary anti-rabbit IgG	Abcam, Cat#ab172730, UK
secondary anti-mouse IgG	Abcam, Cat#ab133470, UK

Supplementary Table 4. Differential gene expression analysis in the GSE70362 dataset stratified by degeneration severity

Gene	logFC#	p.value	Gene	logFC	p.value
IGFBP3	2.51	0.00	TRIM29	-1.00	0.01
COL10A1	2.05	0.02	QDPR	-1.00	0.00
IGFBP1	1.95	0.02	CYB5R2	-1.01	0.00
SMIM3	1.94	0.00	DPT	-1.02	0.02
TFPI	1.83	0.00	RAPGEF5	-1.02	0.01
DEFB1	1.70	0.00	TBXT	-1.02	0.01
GATA6	1.65	0.00	CPAMD8	-1.03	0.00
LMO2	1.63	0.00	SGCE	-1.03	0.00
AKR1C3	1.54	0.02	PALLD	-1.04	0.00
GDF15	1.48	0.00	ACTC1	-1.05	0.04
NQO1	1.47	0.01	CRISPLD2	-1.05	0.01
CCND1	1.42	0.00	CHST10	-1.07	0.00

NANOS1	1.39	0.00	DCXR	-1.08	0.00
ZFP36L1	1.37	0.00	COL6A2	-1.10	0.00
IFIT3	1.37	0.00	AOX1	-1.10	0.00
CYP1B1	1.32	0.00	MT1F	-1.12	0.01
HSD11B1	1.29	0.01	DMKN	-1.13	0.00
EMILIN1	1.28	0.00	TEX14	-1.13	0.00
ZEB2	1.26	0.00	SHISA2	-1.14	0.02
TNFAIP6	1.25	0.00	SCRN1	-1.15	0.00
EBF1	1.24	0.00	LY75	-1.17	0.01
SOX4	1.20	0.01	ENPP2	-1.22	0.00
IFIT2	1.17	0.04	CHMP4C	-1.23	0.01
GREM1	1.17	0.01	NEFM	-1.23	0.03
CYTL1	1.16	0.02	MT1M	-1.24	0.03
MGST1	1.15	0.00	FOXQ1	-1.24	0.01
IFIT1	1.14	0.03	ABLIM1	-1.25	0.01
CNIH3	1.13	0.00	FOXF2	-1.26	0.00
FAT4	1.13	0.01	PDLIM1	-1.29	0.00
SLC16A6	1.12	0.00	LDLR	-1.32	0.00
PTHLH	1.11	0.00	ZNF185	-1.33	0.00
CD1D	1.10	0.03	HAS2	-1.34	0.00
DLX3	1.10	0.00	S100A2	-1.35	0.03
CHI3L1	1.10	0.05	LYVE1	-1.35	0.00
ST6GALNAC2	1.10	0.00	VAMP8	-1.35	0.00
SLITRK4	1.09	0.00	HYAL1	-1.38	0.00
GBP1	1.09	0.01	CNMD	-1.40	0.03
IRX5	1.08	0.01	CLEC3A	-1.44	0.01
CHAC2	1.06	0.04	CLTRN	-1.45	0.00
PTGDS	1.05	0.00	HOPX	-1.57	0.00
RBM20	1.02	0.00	KRT19	-1.61	0.02
RAP1A	1.02	0.02	SDR16C5	-1.62	0.00
DUSP5	1.02	0.01	MT1G	-1.70	0.00
			SCGB2A2	-1.78	0.00
			SCGB1D2	-1.80	0.02
			IBSP	-2.49	0.00

logFC, log (Fold Change).

Supplementary Table 5. 844 ferroptosis-associated genes from the FerrDb V2 database

symbol	confidence	symbol	confidence	symbol	confidence
RPL8	Validated	ATF3	Validated	NEDD4	Validated
IREB2	Validated	HMOX1	Validated	FXN	Validated
ATP5MC3	Validated	HMGB1	Validated	AIFM2	Validated
CS	Validated	EPAS1	Validated	PRDX1	Deduced
EMC2	Validated	SNX5	Validated	AR	Validated
ACSF2	Validated	PAQR3	Validated	CBS	Validated
NOX1	Deduced	MICU1	Validated	NFE2L2	Validated
CYBB	Deduced	NOX4	Validated	CHMP5	Validated
NOX3	Deduced	TOR2A	Predicted	CHMP6	Validated
NOX4	Deduced	MIR375	Validate	HMOX1	Validated
NOX5	Deduced	MAP3K14	Validate	ZFP36	Validated
DUOX1	Deduced	SIRT3	Validate	LAMP2	Validated
DUOX2	Deduced	CircKDM4C	Validated	MTF1	Validated
G6PD	Validated	MIR324	Validated	COPZ1	Validated
PGD	Validated	QSOX1	Validated	NUPR1	Validated
VDAC2	Validated	MIB2	Validated	USP35	Validated

PIK3CA	Validated	CLTRN	Validated	HSF1	Validated
FLT3	Validated	KLF2	Validated	PROM2	Validated
SCP2	Validated	MIR5096	Validated	PLA2G6	Validated
TP53	Validated	TFRC	Validated	HIF1A	Validated
ACSL4	Predicted	HOTAIR	Validated	NEAT1	Validated
LPCAT3	Predicted	H19	Validated	RRM2	Validated
NRAS	Deduced	FOXO4	Deduced	SLC7A11	Validated
KRAS	Deduced	ELAVL1	Validated	FTMT	Validated
HRAS	Deduced	YTHDC2	Validated	PARP1	Deduced
TF	Validated	DDR2	Validated	PARP2	Deduced
TFRC	Validated	SLC39A7	Validated	PARP3	Deduced
TFR2	Validated	TRIM46	Validated	PARP4	Deduced
SLC38A1	Validated	ACSL1	Validated	PARP6	Deduced
SLC1A5	Validated	KDM5A	Validated	PARP8	Deduced
GLS2	Validated	TRIM21	Validated	PARP9	Deduced
GOT1	Validated	HMOX1	Validated	PARP10	Deduced
CARS1	Validated	DPEP1	Validated	PARP11	Deduced
TP53	Validated	CYGB	Validated	PARP12	Deduced
ALOX5	Validated	IDO1	Validated	PARP14	Deduced
KEAP1	Validated	GSTZ1	Validated	PARP15	Deduced
HMOX1	Validated	TP53	Validated	PARP16	Deduced
TP53	Validated	ACO1	Validated	PDSS2	Validated
TP53	Validated	GJA1	Validated	TXN	Validated
GLS2	Validated	IREB2	Validated	SENP1	Validated
ATG5	Validated	SLC7A11	Validated	PLA2G6	Validated
ATG7	Validated	PGRMC1	Validated	OIP5-AS1	Validated
NCOA4	Validated	CIRBP	Validated	MIR190A	Validated
TF	Validated	FAR1	Validated	FGF21	Validated
ALOX5	Validated	circPSEN1	Validated	CREB1	Deduced
ALOX12	Validated	USP11	Validated	CREB3	Deduced
ALOX12B	Validated	STING1	Validated	CREB5	Deduced
ALOX15	Validated	YAP1	Validated	FTMT	Validated
ALOX15B	Validated	HMOX1	Validated	GOT1	Validated
ALOXE3	Validated	MIR135B	Validated	TFRC	Validated
PHKG2	Validated	TRIM26	Validated	GPX4	Validated
TFRC	Screened	YAP1	Validated	MIR130B	Validated
ACO1	Screened	NDRG1	Deduced	BEX1	Validated
IREB2	Screened	MIR302A	Validated	ASAH2	Validated
SLC38A1	Screened	ASMTL-AS1	Validated	SCD	Validated
GLS2	Screened	ZFAS1	Validated	FABP4	Validated
G6PDX	Screened	FADS2	Validated	AKT1S1	Deduced
ULK1	Validated	PIEZO1	Validated	MLST8	Deduced
ATG3	Validated	LIFR	Validated	MTOR	Deduced
ATG4D	Screened	PTPN6	Validated	RPTOR	Deduced
ATG5	Validated	MIR15A	Validated	CDH1	Validated
BECN1	Screened	EGR1	Validated	SIRT1	Validated
MAP1LC3A	Screened	ADAM23	Validated	TYRO3	Validated
GABARAPL2	Screened	ARHGEF26-AS1	Validated	SIRT6	Validated
GABARAPL1	Screened	ACSL4	Validated	TMSB4X	Deduced
ATG16L1	Screened	CPEB1	Validated	TMSB4Y	Deduced
WIPI1	Screened	COX4I2	Validated	KIF20A	Validated
WIPI2	Screened	lncRNA AABR07017145.1	Validated	ECH1	Validated
SNX4	Screened	TIMP1	Validated	circRHOT1	Validated
ATG13	Validated	MIR15A	Validated	ETV4	Validated

ULK2	Validated	KDM6B	Validated	MEG8	Validated
NCOA4	Validated	NCOA4	Validated	VCP	Validated
ACSL4	Validated	GSK3B	Validated	circ_0007142	Validated
TP53	Validated	IFNG	Validated	ENPP2	Validated
SAT1	Validated	METTL14	Validated	RBMS1	Validated
ALOX15	Validated	CHAC1	Validated	KDM4A	Validated
ACSL4	Validated	MIB1	Validated	CBS	Validated
LPCAT3	Validated	KDM5C	Validated	MGST1	Validated
ALOX15	Validated	ACSL4	Validated	circKIF4A	Validated
ACSL4	Validated	MEG3	Validated	miR-7-5p	Validated
KEAP1	Validated	CCDC6	Validated	PRDX6	Validated
EGFR	Validated	ATF3	Validated	circ_0067934	Validated
NOX4	Validated	IREB2	Validated	MPC1	Validated
MAPK3	Validated	CFL1	Validated	CHMP1A	Validated
MAPK1	Validated	ALOXE3	Validated	CAMKK2	Validated
BID	Validated	MIR539	Validated	SOX2	Validated
ACSL4	Validated	KMT2D	Validated	SRSF9	Validated
ZEB1	Validated	SLC7A11	Validated	PROK2	Validated
KEAP1	Validated	GPX4	Validated	MIR4443	Validated
DPP4	Validated	AKR1C1	Validated	SIRT2	Validated
ALOX15	Validated	AKR1C2	Validated	circRNA1615	Validated
ALOX12	Validated	AKR1C3	Validated	MIR27A	Validated
CDKN2A	Validated	GPX4	Validated	MIR670	Validated
PEBP1	Validated	RB1	Validated	MEF2C	Validated
SOCS1	Validated	HSPB1	Validated	NF2	Validated
CDO1	Validated	HSF1	Validated	CDH1	Validated
MYB	Validated	SLC7A11	Validated	HSPB1	Validated
HMOX1	Validated	GPX4	Validated	EZH2	Validated
MAPK8	Validated	GCLC	Validated	PEDS1	Validated
MAPK9	Validated	SLC7A11	Validated	SMPD1	Validated
MAPK1	Validated	NFE2L2	Validated	ADAMTS13	Validated
MAPK3	Validated	SQSTM1	Validated	CDC25A	Validated
SLC1A5	Validated	NQO1	Validated	G6PD	Validated
CHAC1	Validated	HMOX1	Validated	SRSF9	Validated
MAPK14	Validated	FTH1	Validated	CAV1	Validated
LINC00472	Validated	MUC1	Validated	CircFNDC3B	Validated
NOX4	Validated	SLC3A2	Validated	PPARD	Validated
GOT1	Validated	MT1G	Validated	CISD2	Validated
BECN1	Validated	NFE2L2	Deduced	ENO3	Validated
PRKAA2	Validated	SLC40A1	Validated	SESN2	Validated
PRKAA1	Validated	SLC7A11	Validated	LCN2	Validated
ELAVL1	Validated	GPX4	Validated	MARCHF5	Validated
BAP1	Validated	SLC7A11	Validated	TRIB2	Validated
TP53	Validated	CISD1	Validated	DHODH	Validated
ABCC1	Validated	SLC7A11	Validated	SLC7A11	Validated
ACSL4	Validated	FANCD2	Validated	MIR545	Validated
MIR6852	Validated	GPX4	Validated	OTUB1	Validated
ACVR1B	Validated	NFE2L2	Validated	PDK4	Validated
TGFBR1	Validated	FTMT	Validated	CircPVT1	Validated
BAP1	Validated	HSPA5	Validated	MIR9-3HG	Validated
EPAS1	Validated	ATF4	Validated	ADIPOQ	Validated
HILPDA	Validated	SLC7A11	Validated	circDTL	Validated
HIF1A	Validated	GPX4	Validated	GPX4	Validated
ALOX12	Validated	GPX4	Validated	mmu_circRNA_0000309	Validated
ACSL4	Validated	HMOX1	Validated	IL6	Validated

HMOX1	Validated	ATF4	Validated	PTPN18	Validated
IFNG	Validated	NFE2L2	Validated	FTH1	Validated
ANO6	Validated	TP53	Validated	FTH1	Validated
LPIN1	Validated	SLC7A11	Validated	FTL	Validated
HMGB1	Validated	HELLS	Validated	LCN2	Validated
TNFAIP3	Validated	SCD	Validated	ABCC5	Validated
TLR4	Validated	FADS2	Validated	CISD3	Validated
NOX4	Validated	SRC	Validated	MS4A15	Validated
ATF3	Validated	STAT3	Validated	LCN2	Validated
ATM	Validated	NFE2L2	Validated	FURIN	Deduced
YY1AP1	Validated	PML	Validated	circRHBG	Validated
EGLN2	Validated	MTOR	Validated	GALNT14	Validated
MIOX	Validated	NFS1	Validated	KLHDC3	Validated
TAFAZZIN	Validated	TP63	Validated	LINC01833	Validated
MTDH	Validated	SLC7A11	Validated	circGFRA1	Validated
IDH1	Validated	TP53	Validated	MAPKAP1	Deduced
SIRT1	Predicted	CDKN1A	Validated	MLST8	Deduced
TAFAZZIN	Validated	MIR137	Validated	MTOR	Deduced
BECN1	Validated	SLC40A1	Validated	PRR5	Deduced
FBXW7	Deduced	GPX4	Validated	RICTOR	Deduced
PANX1	Validated	GPX4	Deduced	GSTM1	Validated
DNAJB6	Predicted	ENPP2	Validated	TERT	Validated
BACH1	Validated	VDAC2	Validated	circ0097009	Validated
ACSL4	Validated	FH	Validated	TMEM161B-DT	Validated
LONP1	Validated	CISD2	Validated	circEPSTI1	Validated
CD82	Validated	SLC40A1	Validated	MIR18A	Deduced
IL1B	Validated	MIR9-1	Validated	RARRES2	Validated
CTSB	Validated	MIR9-2	Validated	USP11	Validated
POR	Validated	MIR9-3	Validated	PTGS2	Validated
CYB5R1	Validated	CBS	Validated	CHAC1	Validated
ELOVL5	Validated	NFE2L2	Validated	SLC40A1	Validated
FADS1	Validated	SQSTM1	Validated	TF	Validated
ALOX12	Deduced	GPX4	Validated	TFRC	Validated
FBXW7	Validated	ISCU	Validated	FTH1	Validated
PTEN	Deduced	FTH1	Validated	GPX4	Validated
NR1D1	Deduced	ACSL3	Validated	HSPB1	Validated
NR1D2	Deduced	OTUB1	Validated	NFE2L2	Validated
TBK1	Validated	CD44	Validated	GPX4	Validated
IL6	Validated	LINC00336	Validated	FTH1	Validated
USP7	Validated	STAT3	Validated	DUSP1	Deduced
miR-182-5p	Validated	BRD4	Validated	NOS2	Deduced
miR-378a-3p	Validated	PRDX6	Validated	NCF2	Deduced
CTSB	Deduced	MIR17	Validated	MT3	Deduced
ACSL4	Deduced	SCD	Validated	UBC	Deduced
ATF4	Validated	SESN2	Validated	ALB	Deduced
BECN1	Deduced	NF2	Validated	TXNRD1	Deduced
AQP3	Deduced	ARNTL	Validated	SRXN1	Deduced
AQP5	Deduced	HIF1A	Validated	GPX2	Deduced
AQP8	Deduced	JUN	Deduced	BNIP3	Deduced
LINC00618	Validated	CA9	Validated	OXSR1	Deduced
IREB2	Deduced	HSPA5	Validated	SELENOS	Deduced
MT1DP	Validated	TMBIM4	Validated	ANGPTL7	Deduced
ACSL4	Screened	HSPA5	Predicted	SLC7A11	Deduced
PEX10	Validated	PLIN2	Screened	DDIT4	Screened
KEAP1	Screened	MIR212	Validated	LOC284561	Screened

AGPAT3	Screened	Fer1HCH	Predicted	ASNS	Screened
PEX12	Validated	AIFM2	Validated	TSC22D3	Screened
CHP1	Screened	AIFM2	Validated	DDIT3	Screened
GPAT4	Screened	LAMP2	Validated	JDP2	Screened
BRPF1	Screened	ZFP36	Validated	SESN2	Screened
OSBPL9	Screened	GPX4	Validated	SLC1A4	Screened
INTS2	Screened	PROM2	Validated	PCK2	Screened
MMD	Screened	CHMP5	Validated	TXNIP	Screened
CYP4F8	Screened	CHMP6	Validated	VLDLR	Screened
MLLT1	Screened	AKR1C1	Validated	GPT2	Screened
TTPA	Screened	AKR1C2	Validated	PSAT1	Screened
GRIA3	Screened	AKR1C3	Validated	LURAP1L	Screened
EPT1	Screened	CBS	Validated	SLC7A5	Screened
POM121L12	Screened	NFE2L2	Validated	HERPUD1	Screened
LIG3	Screened	CAV1	Validated	XBP1	Screened
AEBP2	Screened	GCH1	Validated	ATF3	Screened
AGPS	Validated	SIRT3	Validated	SLC3A2	Screened
CDCA3	Screened	DAZAP1	Validated	CBS	Screened
PEX2	Validated	PIR	Validated	ATF4	Screened
LPCAT3	Screened	GCLC	Validated	ZNF419	Screened
PEX6	Screened	FTL	Validated	KLHL24	Screened
TIMM9	Screened	HCAR1	Validated	TRIB3	Screened
DCAF7	Screened	SLC16A1	Validated	ZFP69B	Screened
LCE2C	Screened	RRM2	Validated	ATP6V1G2	Screened
FAR1	Validated	SCD	Validated	VEGFA	Screened
PHF21A	Screened	NR4A1	Deduced	GDF15	Screened
SMAD7	Screened	PIK3CA	Deduced	TUBE1	Screened
LYRM1	Screened	RPTOR	Deduced	ARRDC3	Screened
AMN	Screened	SREBF1	Validated	CEBPG	Screened
PEX3	Validated	SREBF2	Validated	SNORA16A	Screened
MTCH1	Screened	FZD7	Validated	RGS4	Screened
ZEB1	Validated	NFE2L2	Validated	BLOC1S5-TXNDC5	Screened
SIRT1	Validated	NFE2L2	Validated	LOC390705	Screened
ACADSB	Deduced	P4HB	Deduced	EIF2S1	Deduced
PVT1	Validated	NT5DC2	Deduced	KIM-1	Deduced
hsa_circ_0008367	Validated	BCAT2	Validated	IL6	Deduced
SLC39A14	Validated	HSF1	Validated	CXCL2	Deduced
NCOA4	Deduced	PLA2G6	Validated	RELA	Deduced
MAP3K11	Validated	MIR424	Validated	HSD17B11	Screened
GSK3B	Validated	PARK7	Validated	AGPAT3	Screened
MAPK8	Validated	FXN	Validated	SETD1B	Screened
BRD7	Validated	SUV39H1	Validated	HMOX1	Deduced
TP53	Validated	ATF2	Validated	TF	Deduced
SLC25A28	Validated	CDKN1A	Predicted	FTL	Deduced
ACSL4	Validated	FTH1	Validated	RPL8	Deduced
MFN2	Validated	NFE2L2	Validated	ATP5MC3	Deduced
ACSL4	Validated	STAT3	Validated	TFRC	Deduced
SLC11A2	Validated	ACOT1	Validated	MAFG	Deduced
ZFAS1	Validated	NFE2L2	Validated	IL33	Deduced
SLC38A1	Deduced	ALDH3A2	Validated	FTH1	Validated
TSC1	Validated	NFE2L2	Validated	HAMP	Deduced
PEBP1	Validated	STK11	Validated	STEAP3	Deduced
TGFB1	Deduced	FNDC5	Deduced	DRD5	Deduced
SNCA	Validated	CircIL4R	Validated	GPX4	Deduced
SIRT3	Validated	CDH1	Validated	DRD4	Deduced

PRKAA2	Validated	NFE2L2	Validated	MAP3K5	Deduced
TFRC	Validated	MIR214	Validated	MAPK14	Deduced
CGAS	Validated	NEDD4L	Validated	SLC2A1	Deduced
STING1	Validated	SQSTM1	Validated	SLC2A3	Deduced
HDDC3	Validated	TF	Validated	SLC2A6	Deduced
MIR761	Validated	FTMT	Validated	SLC2A8	Deduced
MDM2	Validated	BRD2	Deduced	SLC2A12	Deduced
MDM4	Validated	BRD3	Deduced	GLUT13	Deduced
ALOX15	Validated	BRD4	Deduced	SLC2A14	Deduced
POR	Validated	BRDT	Deduced	EIF2AK4	Deduced
MIR214	Validated	SCD	Validated	EIF2S1	Deduced
DLD	Validated	SLC7A11	Validated	ATF4	Deduced
LONP1	Validated	DECR1	Validated	ALOX5	Deduced
ACSL4	Validated	NFE2L2	Validated	ALOX12	Deduced
BACH1	Validated	GPX4	Validated	ALOX15	Deduced
DNAJB6	Validated	SLC7A11	Validated	ALOX5	Deduced
WWTR1	Validated	NFE2L2	Validated	ACSF2	Deduced
SIRT1	Validated	GLRX5	Validated	IREB2	Deduced
ATM	Validated	GPX4	Validated	HMGB1	Deduced
PRKCA	Validated	NCOA3	Deduced	HMOX1	Deduced
LGMN	Validated	NR5A2	Deduced	NFE2L2	Deduced
ACSL4	Validated	GPX4	Validated	ELAVL1	Deduced
TP53	Validated	MTOR	Deduced	SLC3A2	Deduced
IFNG	Validated	PANX2	Validated	SLC7A11	Deduced
SMPD1	Validated	RHEBP1	Validated	TFAP2C	Deduced
MYCN	Validated	TFAP2A	Validated	SP1	Deduced
SLC11A2	Validated	CP	Validated	HBA1	Deduced
IFNA1	Deduced	SLC7A11	Validated	NNMT	Deduced
IFNA2	Deduced	ARF6	Validated	PLIN4	Deduced
IFNA4	Deduced	GDF15	Validated	HIC1	Deduced
IFNA5	Deduced	ABHD12	Validated	STMN1	Deduced
IFNA6	Deduced	PPP1R13L	Validated	RRM2	Deduced
IFNA7	Deduced	TFAM	Validated	CAPG	Deduced
IFNA8	Deduced	KDM3B	Validated	HNF4A	Deduced
IFNA10	Deduced	RNF113A	Validated	NGB	Deduced
IFNA13	Deduced	PARK7	Validated	YWHAE	Deduced
IFNA14	Deduced	AHCY	Deduced	GABPB1	Deduced
IFNA16	Deduced	FXN	Validated	AURKA	Predicted
IFNA17	Deduced	circ-TTBK2	Validated	MIR4715	Predicted
IFNA21	Deduced	MIR522	Validated	RIPK1	Deduced
SMG9	Validated	IDH2	Validated	PRDX1	Deduced
NR1D1	Validated	PPARA	Validated	MIR30B	Deduced
ACSL4	Validated	NOS2	Validated	MMP13	Deduced
PPARG	Validated	SIAH2	Validated	LRRFIP1	Deduced
TLR4	Validated	RELA	Validated	CBR1	Deduced
IL6	Validated	PRKAA2	Validated		
MIR335	Validated	VDR	Validated		

Supplementary Table 6. The LASSO coefficients in the combined dataset of GSE70362

Variable	λ (min)
(Intercept)	8.29557083

ENPP2	-1.3422454
CLTRN	0
MGST1	0
MT1G	-0.1589492
GDF15	0
NQO1	0
AKR1C3	0.40907799

Supplementary Table 7. The correlation between risk score and genes with a threshold of |cor| > 0.75 and p < 0.05 in GSE70362

risk_score	Gene	cor	p.value
risk_score	ENPP2	-0.91276	5.11E-10
risk_score	PTRHD1	-0.86681	4.3E-08
risk_score	ZDHHC9	-0.85744	8.67E-08
risk_score	CHMP4C	-0.83135	4.85E-07
risk_score	MAP3K21	-0.81842	1.02E-06
risk_score	PRKCZ	-0.81369	1.33E-06
risk_score	SGCE	-0.80995	1.62E-06
risk_score	LGALSL	-0.80287	2.34E-06
risk_score	NGEF	-0.80045	2.65E-06
risk_score	HYAL1	-0.79866	2.89E-06
risk_score	PROK2	-0.79789	3.01E-06
risk_score	CRYL1	-0.79688	3.16E-06
risk_score	SCGB2A2	-0.79441	3.56E-06
risk_score	RGS3	-0.79005	4.39E-06
risk_score	TRIM29	-0.78755	4.94E-06
risk_score	DLG3	-0.78701	5.07E-06
risk_score	TOP1MT	-0.78652	5.18E-06
risk_score	HEXA	-0.78282	6.14E-06
risk_score	LINC00467	-0.78219	6.33E-06
risk_score	RFC3	-0.77959	7.11E-06
risk_score	MTMR11	-0.77801	7.63E-06
risk_score	CLDN11	-0.77248	9.73E-06
risk_score	C1orf94	-0.77046	1.06E-05
risk_score	IL17RD	-0.76714	1.22E-05
risk_score	SERTAD4-AS1	-0.75828	1.76E-05
risk_score	ARPP19	-0.75707	1.85E-05
risk_score	FOXF2	-0.7556	1.96E-05
risk_score	PERP	-0.75553	1.97E-05
risk_score	NFYC	-0.75489	2.02E-05
risk_score	LYVE1	-0.75383	2.1E-05
risk_score	TIAM1	-0.75287	2.19E-05
risk_score	POLR1E	-0.75018	2.43E-05
risk_score	SYNJ1	0.750814	2.37E-05
risk_score	ATP11A	0.754378	2.06E-05
risk_score	GLRX	0.756165	1.92E-05
risk_score	USP4	0.75728	1.83E-05
risk_score	EPS8	0.758289	1.76E-05
risk_score	AKR1C3	0.75853	1.74E-05
risk_score	RPN1	0.761806	1.53E-05
risk_score	DSE	0.763675	1.41E-05
risk_score	ERGIC2	0.766093	1.28E-05

risk_score	ZMAT3	0.770368	1.07E-05
risk_score	SMIM3	0.777663	7.75E-06
risk_score	GATA6	0.78803	4.83E-06
risk_score	SLC40A1	0.795435	3.39E-06

Supplementary Table 8. Differential gene expression analysis in the GSE70362 dataset stratified by GATA6

Gene	logFC	pValue	Gene	logFC	pValue
IGFBP3	2.76	0.00	FOXF2	-1.01	0.00
IGFBP1	2.28	0.00	GSTO2	-1.01	0.00
MMP2	1.91	0.02	COL9A2	-1.03	0.01
AKR1C3	1.84	0.00	H4C8	-1.03	0.02
SPARCL1	1.76	0.02	MT1F	-1.03	0.01
GATA6	1.76	0.00	PDLIM1	-1.04	0.01
SMIM3	1.65	0.00	COL11A2	-1.04	0.01
MMP1	1.61	0.01	CKB	-1.05	0.00
TFPI	1.55	0.00	ADTRP	-1.07	0.00
NQO1	1.52	0.00	ZNF185	-1.07	0.00
CD1D	1.40	0.00	MYOT	-1.08	0.01
ADAMTS5	1.35	0.02	VAMP8	-1.08	0.01
ZEB2	1.34	0.00	HAS2	-1.09	0.01
IRX3	1.28	0.01	DCXR	-1.10	0.00
EBF1	1.28	0.00	RGS16	-1.10	0.01
GDF15	1.25	0.01	FOXQ1	-1.12	0.01
RIN2	1.22	0.01	CLTRN	-1.15	0.00
FAT4	1.21	0.00	SPON2	-1.15	0.01
COL4A1	1.16	0.01	ENPP2	-1.20	0.00
LMO2	1.15	0.00	HOPX	-1.21	0.02
CYP1B1	1.14	0.00	CLEC3A	-1.22	0.03
FZD10	1.12	0.04	TRIM29	-1.23	0.00
ALCAM	1.09	0.00	MT1G	-1.27	0.01
MAN1A1	1.09	0.01	WIF1	-1.29	0.04
ALDH3A2	1.08	0.00	SHISA2	-1.30	0.00
CD14	1.04	0.04	SDR16C5	-1.30	0.01
MGST1	1.04	0.00	CNMD	-1.30	0.03
RERG	1.04	0.02	CHRDL2	-1.37	0.05
CHAC2	1.03	0.04	ZNF165	-1.37	0.03
SLC40A1	1.02	0.00	CHMP4C	-1.40	0.00
DMXL2	1.01	0.00	KRT19	-1.48	0.02
PXDN	1.01	0.00	NEFM	-1.54	0.00
CCND1	1.01	0.01	IBSP	-1.86	0.01
HSD11B1	1.00	0.03	SCGB2A2	-1.87	0.00
			SCGB1D2	-1.94	0.00

Supplementary Table 9. The GSEA results in GSE70362 using differentially expressed genes stratified by GATA6 expression

Description	setSize	enrichmentScore	NES	pvalue	p.adjust

Steroid hormone biosynthesis	47	0.67	2.14	5.37E-06	0.000913
Bladder cancer	41	0.61	1.91	0.000624	0.017678
Proteasome	42	0.59	1.84	0.001269	0.030809
N-Glycan biosynthesis	50	0.58	1.88	0.000349	0.013586
Malaria	46	0.57	1.81	0.001741	0.031148
Arachidonic acid metabolism	54	0.54	1.77	0.00314	0.044481
Protein digestion and absorption	94	0.51	1.84	0.000106	0.009267
Toll-like receptor signaling pathway	96	0.50	1.81	0.000306	0.013586
AGE-RAGE signaling pathway in diabetic complications	99	0.48	1.75	0.000411	0.013962
Amoebiasis	98	0.46	1.67	0.001571	0.031148
MicroRNAs in cancer	158	0.44	1.70	0.000132	0.009267
Fluid shear stress and atherosclerosis	129	0.43	1.63	0.001698	0.031148
Protein processing in endoplasmic reticulum	161	0.42	1.63	0.00036	0.013586
Alcoholic liver disease	132	0.42	1.60	0.002169	0.033515
Relaxin signaling pathway	125	0.42	1.59	0.001961	0.03175
NOD-like receptor signaling pathway	166	0.41	1.59	0.000459	0.014181
Cellular senescence	153	0.40	1.56	0.001941	0.03175
Proteoglycans in cancer	193	0.40	1.57	0.000969	0.025356
Lipid and atherosclerosis	201	0.39	1.57	0.000228	0.01294
Herpes simplex virus 1 infection	429	0.38	1.64	4.58E-07	0.000156
Alzheimer disease	345	0.33	1.41	0.0016	0.031148
Human papillomavirus infection	315	0.33	1.36	0.002368	0.034999
Alcoholism	147	-0.48	-1.71	0.000136	0.009267
Amphetamine addiction	68	-0.54	-1.72	0.001364	0.030918
Cocaine addiction	48	-0.55	-1.65	0.003562	0.048448
